# Starting, staying and succeeding: motivation, life aspirations and hobbies as possible predictors of progression through a real-world online weight loss program

**DOI:** 10.1186/s12889-026-28368-z

**Published:** 2026-07-06

**Authors:** Emily Brindal, Paige G Brooker, Gilly A Hendrie

**Affiliations:** CSIRO Health & Biosecurity, Gate 13 Kintore Avenue, Adelaide, SA 5000 Australia

**Keywords:** Motivation, Self-determination, Weight loss, Aspirations, Behavioural initiation

## Abstract

**Background:**

Autonomous motivation is widely considered important for behaviour change, particularly around longer term, goal-orientated pursuits such as weight management. Few studies have explored how motivation predicts progression in real-world weight management from initiation to weight loss.

**Methods:**

The Diet Mindset Quiz was developed as a health promotion tool that captures aspects of motivation based on self-determination theory, as well as broader life aspirations and intrinsic motivators or hobbies. The Quiz is provided free on the CSIRO Total Wellbeing Diet (TWD) platform – which hosts the structured 12-week online weight loss program. The current analysis linked data from the Quiz with data from the TWD program to understand how motivational characteristics predicted three stages of progress in weight management: initiation (signing up), early behavioural commitment (staying for the full 12-week program) and early success (weight loss outcome). Regression analyses were used to compare those who started the TWD after completing the Diet Mindset Quiz and those who remained on the TWD for 12 weeks and the association between individual and motivational characteristics and weight loss.

**Results:**

The final dataset included 98,335 Australians classified as overweight or obese based on self-reported height and weight. The sample was mostly female (83.4%) and aged 51–70 years (48.4%). Of those who took the quiz, 11.1% (*n* = 10,808) went on to sign up to the TWD program and 5.3% (*n* = 5139) stayed on the program for the full 12-weeks. Overall, the models predicted small amounts of the stages considered (1–5%). Intrinsic life aspirations increased the probability of signing up to the TWD by 26%. Mentally-orientated hobbies (e.g., puzzles, brain training games) increased probability of remaining for 12 weeks by 32%. Greater autonomous and introjected motivation had very small association with an increased probability of initiating the program, but lower weight loss at 12 weeks.

**Conclusions:**

Predictive value of motivational constructs for stages of progression in weight loss was minimal. Within these, the small effects for autonomous motivation suggest a lack of practical implication. The measurement and timing of assessing motivation requires a skilful brush. Perhaps it is less important to understand initial motivations than to purposefully develop them.

## Background

Self-Determination Theory (SDT) is a well-established framework for explaining the process of motivation and the circumstances that promote effort, commitment, mastery and behavioural regulation [1]. Motivation is understandably important in the context of health behaviours and the pursuit of lifestyle improvements, which often involve challenging behaviours, reprogramming of habits and the requirement for sustained change. Meta-analysis has been used to broadly support the application of SDT to health behaviour [2].

According to SDT, the satisfaction of three basic needs (autonomy, competence and relatedness) can promote optimal functioning, growth, social development and wellbeing [3]. SDT also attempts to explain which motivational characteristics move a person from intention to action [3]; a significant gap in the process of health behaviour change [4]. Within SDT, intrinsic motivation (i.e., doing things for fun, and the desire to learn and grow) represents the form of motivation that can be considered most pure and potent because it leverages natural drive [1] rather than relying on self-control – which can become depleted over time [5]. Importantly, intrinsic motivation is said to underpin autonomous motivation (AM). It is theorised that a greater sense of choice to perform, greater interest and higher satisfaction can result in greater persistence in the face of challenges or fatigue [6].

SDT [1] also identifies extrinsic motivation which is underpinned by four types of regulation: external (i.e., involving rewards), introjected (i.e., internal rewards and self-control), identified (i.e., personal importance), and integrated (i.e., self-congruency) [7]. Identified and integrated regulation are internal versions of extrinsic motivation. A further layer that has been added to SDT is the concept of controlled motivation which some authors have argued is more important than the distinction between intrinsic and extrinsic [8]. Controlled motivation (CM) encompasses motivations underpinned by a contingency to receive something (external) or avoid something negative (introjected). In contrast, AM is considered internally focused, driven by personal values rather than external contingencies.

While SDT contains detailed definitions for these varied forms of motivation, the theory does not provide validated means of measuring motivation-associated constructs. Some investigators have scored AM and CM separately, others have combined the two to create a single score, and some have considered individual types of motivation [9]. For example, research examining motivational profiles rather than pure levels of motivation highlights the co-existence between controlled and autonomous motivation for students’ academic outcomes with those displaying both performing better [8]. Other studies that have explored individual types of motivation have also suggested these independently contribute to lifestyle behaviours in the examples of physical challenges [10], sustained eating behaviours [11], and action in physical pursuits [12]. With no clear standards for measurement, it is difficult to understand how and why aspects of motivation may improve behaviour.

Weight management is a particularly relevant context for examining motivational dynamics. It requires the simultaneous regulation of multiple health behaviours, including dietary intake, physical activity, and program engagement, over sustained periods, and is characterised by high rates of attrition and relapse. Weight management also involves navigating stages of change from initial intention through to sustained action, each of which may be driven by different motivational characteristics. Yet, most research has focussed on the role of motivation only during active weight loss. A review of psychosocial pre-treatment predictors of weight loss outcomes found that an autonomous, self-motivated cognitive style was among the most consistent prospective predictor of weight management success, yet even the strongest predictive models explained only 20–35% of the variance in weight change [13]. A subsequent systematic review of self-regulation mediators in obesity interventions identified autonomous motivation, alongside self-efficacy and self-regulation skills, most consistently linked to beneficial weight and physical activity outcomes [14]. Other interventional studies have also generally supported the idea that levels of AM at varying stages of active intervention could improve outcomes and goal progress in weight management [15]. Yet, existing studies have included motivated participants, actively attempting to control their weight. Almost all (65/66) participants in the study by Webber et al. [15] completed the full weight loss program. Staying on a weight management program is a motivational challenge, but beginning a program is an equally important, yet understudied, stage.

Beyond motivation specifically relating to lifestyle behaviours and weight management, a person has broader motivational drivers and aspirations that may be leveraged to improve progress and achievement, particularly when intervention behaviours themselves may not provide intrinsic reward. Regularly, motivation has been measured using formats that define a behaviour directly associated with a targeted goal (e.g., I am trying to improve my diet because…) and then ask participants to rate underlying reasons based on SDT motivations [16]. This approach could be confounded by behavioural characteristics. For example, consuming discretionary foods (or “junk foods”) and eating with other people can both contribute to greater energy intake and excess weight [17] but also provide some intrinsic pleasure through hedonism and social connection. When asked directly about motivational reasons for ‘eating less junk food’, AM may be low. In this context, broader aspirations and motivations may be more important than those tided specifically to the behaviour. For example, the person may aspire to be healthier which becomes a stronger predictor of their action then the motivation tied specifically to the behaviour. Kasser and Ryan [18] assessed broader life spirations to explore how motivational priorities are linked to greater feelings of vitality. But few have explored how they associate with behaviours and goal pursuits tied to weight management.

Hobbies are an excellent proxy for intrinsic motivation because they are activities that people undertake voluntarily, routinely and generally for fun, which means theoretically they capture AM. Some common hobbies are also associated with positive weight management behaviours – such as exercise or cooking. Being intrinsically interested in positive weight management behaviours could therefore theoretically make some aspects of weight management easier. Yet, limited research has specifically considered the predictive value of broader motivational context and interests simultaneously to different types of motivation.

Studies exploring motivation in active weight management stages are generally supportive of the important role of motivation in weight loss success and early commitment, however, few studies have simultaneously explored how motivation is associated with progression across stages including initiation through to weight loss. Even fewer have considered the role of motivation within broader motivational contexts. The aim of this study was to assess the role of motivation, life aspirations and intrinsic interests (or hobbies) in predicting progression into and through a real-world, structured 12-week weight loss program.

## Methods

The CSIRO Total Wellbeing Diet (TWD) is a structured online weight loss program built around the foundation of a high protein diet. The TWD platform contains menu plans, self-monitoring tools like a food diary and weight tracker, recipes and educational content. As part of the program, various health-related surveys are developed and launched on the program’s webpage to engage current members and attract new members. In January 2021, an online survey was launched that assessed individuals’ autonomous motivation, life aspirations and hobbies – it was called the “Diet Mindset Quiz” [19] (described further below). In 2026, the Quiz remained freely available via the TWD website. At the time of launch the Quiz was promoted through a communications campaign that included free-to-air television, radio and print coverage.

The current study used data collected from the Diet Mindset Quiz between December 2020 and March 2022. The data was provided by Digital Wellness, a company that has an ongoing partnership with CSIRO to deliver the TWD program and manage the platform. All people who start a quiz on the platform agree to terms and conditions including the use of their data for research purposes. Users are assigned a unique identification number which allows their data to be linked across different tools within the platform. All Diet Mindset Quiz participants were sampled through non-probability recruitment methods and self-selected. Given the positioning of the Quiz with communications about health and weight loss, selection may have been biased towards those with greater interest in these areas. This bias applies generally to all data collected on the TWD platform which tends to attract samples with high proportions of middle-aged women [20].

A sample of those who completed the Diet Mindset Quiz were also identified as members of the TWD program. The current analysis used linked data from the Diet Mindset Quiz and the TWD program to understand how motivational characteristics measured in the quiz predicted three discrete stages of progress in weight management – initiation (signing up to the program which involved up-front payment), early behavioural commitment (staying for the initial 12-week program) and early success (weight loss outcome). These stages were defined by available observed data. Only people who qualified for the weight loss program (classified as overweight or obese) and who were not current and former program members were linked to the Diet Mindset Quiz data and included in the analysed data. Behavioural commitment was defined as those who signed up and stayed engaged through to 12 weeks. These people were referred to as “stayers”. Generally, 52% of users are active, at some level by week 12 of the program [20].

Digital Wellness was responsible for the linking the data and provided a merged dataset with deidentified data from the Diet Mindset Quiz and TWD program including a linking code. Program data included sign up date, completion status and self-reported weight loss over 12-weeks and postcode. Socio-Economic Indexes for Areas data collected by the Australian Bureau of Statistics for the Australian Census in 2021 were used to convert postcode into to Relative Socio-economic Disadvantage deciles [21]. The authors were responsible for data cleaning, screening and analysis. Initial data linking and analysis were approved by the CSIRO Low-Risk Human Research Ethics Committee (2022_023_LR), the re-analysis of these data suitable for publication was approved by the same committee under a secondary analysis application (H-2026-0347) all in accordance with the Declaration of Helsinki. Prior to undertaking the surveys or when registering for the TWD, all users provide informed consent to their data being used for research purposes via a digital check box.

### Diet mindset quiz survey items

The final survey items were purposefully selected and tested through a pilot study performed in late 2020, approved under a separate ethics application by CSIRO Low-Risk Human Research Ethics Committee (020_087_LR). In this pilot 953 Australians, mostly female (71.9%) and aged between 18 and 86 (M 54.70, SD 14.85) completed an online survey. These users digitally provided their informed consent to participate after being shown study information. Refinements to some items were made based on this pilot study and are outlined briefly in the appropriate sections below. As well as capturing data, the Quiz provides users with a summary of their motivational profile to promote reflection on their personal drivers.

#### Autonomous motivation

Individuals’ autonomous motivation was captured based on a previously validated questionnaire [16]. Piloting reduced nine possible lifestyle behaviours associated with weight management down to three: Managing weight, eating more healthy foods, being more active. In line with previous administration, participants were asked to rate the reason for pursuing these goals based on the 5 regulatory motivations proposed by SDT (intrinsic, internal, identified, introjected, external) representing aspects of autonomous motivation. Amotivation was also included, “I have no idea why or don’t care”. Given lack of consistency in treatment of motivation score, each motivational type was included as a distinct variable in factor analysis prior to being analysed to understand its structure (described below).

#### Life aspirations

To understand the broader context of a person’s motivational priorities, a list of general aspirations capturing external and intrinsic drives was presented in the survey [18]. In piloting, participants were shown eight items and given the option to enter their own aspirations in free text. Items were refined based on pilot data and this resulted in a list of ten items representing intrinsic (self-acceptance, purpose, connections with others, health, happiness) and extrinsic (popularity, attractiveness, image, career, status) life aspirations. People could choose three aspirations which were then scored into whether they selected all intrinsic aspirations (4), mostly intrinsic (3), mostly external (2), or all external aspirations (1).

#### Intrinsic motivators/hobbies

In piloting, participants were asked to identify an activity they “*do out of pure enjoyment or interest”*. Responses were thematically coded in several passes into 16 common hobbies. The 16 hobbies were then grouped into eight categories based on broader themes: connection, sedentary pastimes, food-related, active, reading/listening, travel, art/creative, and brain training.

#### Demographics

Participants entered their birth year, identified gender, height and weight.

### Analysis

#### Data cleaning

All weight data were cleaned using a standard protocol where extreme Body Mass Index (BMI) (less than 13 kg/m^2^ or greater than 97 kg/m^2^), extreme height (less than 1 m or greater than 3 m) and weight (less than 13 kg or greater than 250 kg) cases were removed. Weight change data was removed if considered extreme when exploring the interquartile ranges (gain of more than 18.8 kg, loss of more than 11.3 kg in 12 weeks). Weight loss was calculated as the percentage of weight lost based on starting body weight. Cases with extreme age (less than 18 or greater than 100 years) or incomplete surveys were also removed. Cleaning resulted in removal of 1% of all original data. Given the study’s focus on weight loss, the sample was restricted to those living with overweight or obesity according to BMI. To simplify interpretation of data in terms of initiation of the program, only people who were not former or current members of the TWD were included in the analysis.

For the stage of behavioural commitment, stayers were defined as members who had at 12 weeks of paid membership and a weight entered in the system at 12 weeks (+/- 1 week). Individuals not meeting these criteria were considered starters – and assumed to have not completed the 12-week program. This protocol has been used previously to define this group and has clear associations with greater weight loss [20].

Given the lack of consistency in the analysis of the motivation scales, factor analysis was used to understand how different types of motivation may be best combined. Separate Factor Analyses using Maximum Likelihood extraction with Promax rotation were performed for each of the domains. All domains showed a tidy 2-factor structure with internal and intrinsic motivation loading together and external and amotivation loading onto a second factor (Table 1). However, loadings for the second factor were not strong (< 0.5) [22].

**Table 1:** Factor analysis loadings for motivational items based on Maximum Likelihood with Promax rotation across three domains of behaviour (*N* = 98,335)

Reason for pursuing goal	Managing Weight		Eating More Healthy Foods		Being More Active	
Factors	1	2	1	2	1	2
Somebody else wants me to or I’ll get something if I do		0.79		0.82		0.82
I would feel ashamed, guilty or anxious if I didn’t		0.33		0.37		0.37
I really believe it is an important goal to have	0.65		0.65		0.71	
It reflects who I am or what I believe in	0.79		0.83		0.84	
It will be fun and enjoyable	0.49		0.62		0.72	
I have no idea why or don’t care		0.35		0.39		0.37

Based on these observations, scores for items in Factor 1 were summed to represent AM, and external and introjected and amotivation scores were retained separately rather than combined into a form of controlled motivation. Consequently, scores for integrated, identified and intrinsic motivation were summed and then divided by 3 to represent the average AM within each domain with scores ranging from a possible 3 to 21 and higher values representing greater AM. Whereas the other motivations were used based on their original scoring between 1 and 7. Bivariate associations between motivations and domains and sensitivity analyses suggested multicollinearity when all behaviours were included separately, therefore, the average of scores across each (weight, eating and activity) were used to represent total motivation values for autonomous (combined), external, introjected, and amotivation across the domains of weight management, eating more healthy foods and increasing activity.

#### Statistical analysis

Motivational items were correlated with a variety of participant characteristics using Pearson Correlation to understand possible relationships.

To assess the core research questions, three predictive models were created. All models included AM, and external, introjected and amotivation scores, the participation (versus not) in eight types of hobbies, and a total intrinsic aspiration score. Due to a skew toward purely intrinsic aspirations, the latter was entered dichotomously representing people with purely intrinsic broader aspirations versus not. In addition to motivational variables, age (years), gender (male or female only), BMI and lifetime diet attempts were controlled for. In models including TWD members, deciles of disadvantage based on residential postcode was also entered. Logistic binary regressions were used to predict signing up to the TWD versus not (initiation) and staying on the program versus not (early behavioural commitment). Finally, linear regression was used to predict percentage weight loss over the 12-week period (early success) based on the user entered data in the platform. In all models, cases any missing data were excluded listwise. After data cleaning, missing data only applied to the SEFIA values due to incomplete or unmatched postcodes.

Given the large sample size, effect sizes (odds ratio and standardised beta values) were used in combination with p levels to identify meaningful results.

## Results

The original response to the Diet Mindset Quiz included 128,604 Australians (between December 2020 and March 2022). A total of 30,274 responses were removed from the sample for this analysis: based on data cleaning (*n* = 119), being previous or current TWD members at the time of the survey (*n* = 11,791) or having a BMI < 25 kg/m^2^ (*n* = 18,359). Of the remaining 98,335 people who completed the Diet Mindset Quiz, 11.1% (*n* = 10,885) went on to sign up to the TWD program and of those who signed up, 5,180 (of 10,885; 47.6%) went on to complete the full 12-week program (stayers). All samples had overrepresentation of females mostly aged between 51 and 70 years and with between 1 and 5 previous attempts to manage their weight (Table 2). Those who stayed on the program were slightly older than the average of the group who signed up, with a slightly greater proportion aged 51 to 70 years (54.6% vs. 52.2%), and a slightly lower mean BMI (32.89 vs. 33.04) but were similarly distributed across previous weight management attempt categories. Greater than 30% of the samples that started the TWD were in the highest deciles for SEIFA (i.e., least disadvantaged).

**Table 2:** Sample descriptive information for each of analysis datasets

Variable		Starting sample	Initiation of a weight management program	Early behavioural commitment
		Completed the Quiz *N* = 98,335 (100%)	Signed up to program *N* = 10,885	Finished 12-week program *N* = 5180
Gender	Male	16,346(16.6)	1,502 (13.8)	750 (14.5)
	Female	81,989(83.4)	9,383 (86.2)	4,430 (85.5)
Age group	18–30 years	7,457 (7.6)	487 (4.5)	176 (3.4)
	31–50 years	37,680 (38.3)	4,155 (38.1)	1,864 (36.0)
	51–70 years	47,593 (48.4)	5,683 (52.2)	2,845 (54.6)
	71 years+	5605 (5.7)	560 (5.1)	295 (5.7)
Body Mass Index (kg/m^2^)	Mean (SD)	33.08 (6.77)	33.04 (6.20)	32.89 (6.26)
Attempts to manage weight	Never	3,501 (3.6)	224 (2.1)	119 (2.3)
	1–5 times	39,546 (40.2)	4,175 (38.3)	2,046 (39.5)
	6–10 times	22,618 (23.0)	2,645 (24.3)	1,283 (24.8)
	11–15 times	9,935 (10.1)	1,244 (11.4)	578 (11.2)
	16–25 times	6,294 (6.4)	773 (7.1)	338 (6.5)
	More than 25 times	16,4541 (16.7)	1,824 (16.8)	816 (15.8)
SEIFA deciles	1	-	412 (3.8)	175 (3.4)
	2	-	637 (5.9)	308 (65.9)
	3	-	735 (6.8)	339 (6.5)
	4	-	850 (7.8)	402 (7.8)
	5	-	1144 (10.5)	546 (10.5)
	6	-	1031 (9.5)	485 (9.4)
	7	-	1010 (9.3)	476 (9.2)
	8	-	1621 (14.9)	774 (14.9)
	9	-	1600 (14.7)	775 (15.0)
	10	-	1768 (16.2)	859 (16.6)
	Missing		77 (0.7)	41 (0.9)

Reported life aspirations were classified as mostly internal/intrinsic by 77.5% of the starting sample (*n* = 76,169). The most common life aspirations selected were health, happiness, and self-acceptance. The most popular hobbies selected by 80.6% of the sample (*n* = 79,279) were those that involved connecting with others, nature, pets or oneself (meditation, relaxation). This was followed by sedentary pursuits (73.5%, *n* = 72,301) and food-based activities (61.0%, *n* = 59,937). Mental hobbies were selected by the smallest proportion of the sample (19.9%, *n* = 19,594).

Motivation scores were skewed toward more favourable profiles with average scores higher than the midpoint for AM and below the midpoint for introjected and amotivation levels (Table 3). Introjected motivation level was positively associated with AM and external motivation. External motivation was weakly to moderately positively associated with amotivation and negatively associated with AM. Weak correlation values (*r* ~ .10) were found between AM and BMI (negative) and number of total hobbies selected. Introjected motivation style had a moderate negative correlation with age.

**Table 3:** Descriptive summary data and correlations (Pearson’s R) for motivation items and other characteristics (*n* = 98,335). All correlation values significant *P*<.001, unless indicated ns

	Pearson’s *R*						
	Range/metric	Mean	SD	AM	Intro	Ex	Amot
*Motivation scores*							
Autonomous (AM)	3 to 21	14.91	3.10	1			
Introjected (Intro)	1 to 7	2.25	1.49	0.23	1		
External (Ex)	1 to 7	3.95	1.67	0.02	0.34	1	
Amotivation (Amot)	1 to 7	1.89	1.20	− 0.22	0.06	0.34	1
*Other characteristics*							
Age	year	51.93	13.04	− 0.03	− 0.28	− 0.06	0.07
Body Mass Index	kg/m^2^	33.08	6.77	− 0.13	0.07	0.10	0.09
Lifetime Dieting frequency	1 to 6	2.26	1.53	0.01	0.08	− 0.01	0.01
Intrinsic Aspirational balance	0 to 3	2.75	0.49	< .01^ns^	− 0.15	− 0.13	− 0.07
Number of Hobbies selected	1 to 4	3.63	0.74	0.12	< .01^ns^	− 0.02	− 0.08

*NS* not significant

### Initiating a weight management program (signing up)

Of those who did the Diet Mindset Quiz, 10,885 people (11.1%) went on to sign up and join the TWD program.

Regression analysis indicated that the 17 variables explained 1% of the variance in signing up to the program (Table 4). Being intrinsically driven increased the probability of signing up by 26%. Five of the hobby categories had a small positive effect on signing up (connection, art/creative, food, reading and travel). Effects for AM and introjected motivation were small and the positive direction.

### Early behavioural commitment (staying for the full 12-week program)

Of those who signed up, 5,180 stayed on the program through to 12 weeks (47.6% of those who signed up; 5.3% of those in original data). The logistic regression model explained 1.5% of the variance for being a Stayer (Table 4). Mentally-orientated hobbies increased probability of completing the program by 32%. Other hobbies (arty, travel reading, and active) also contributed an increased probability of being a classified as a stayer. Having selected only intrinsic life aspirations was the only motivational variable contributing to early commitment with an OR of 1.15.

### Early success (weight loss outcome)

Predictors of weight loss were examined among those who completed the TWD program (*n* = 5180) using hierarchical regression analysis (Table 4). The model accounted for 5.0% of total variance for percentage weight loss, with gender and age the strongest predictors. The motivational variables contributed small effects with greater AM and introjected motivation associated with lower weight loss. Lifetime diet attempts also had a small negative effect with higher attempts resulting in lower percentage body weight loss.

**Table 4:** Model values, odds ratios (OR) and beta values and significant levels (*p*) for regressions predicting initiation, behavioural commitment and early success

Stage	Initiation					Early behavioural commitment					Early success		
Group	Signed up to TWD *N* = 10,885/98,335					Lasted 12 weeks on TWD *N* = 5139/10,808					Percentage weight lost *N* = 5139		
Total variance	Cox and Snell 1.0%					Cox and Snell 1.5%					Adjusted R^2^= 5.0%		
Variables	Wald	*p*	OR	Lower 95% C.I.	Upper 95% C.I.	Wald	*p*	OR	Lower 95% C.I.	Upper 95% C.I.	Beta	t	*p*
Constant	1099.31	< 0.001	0.02	-	-	5.99	0.016	0.57	-	-	-	12.18	< 0.001
Age (years)	119.35	< 0.001	1.01	1.01	1.01	41.35	< 0.001	1.01	1.01	1.02	0.12	7.69	< 0.001
Gender (female)	41.48	< 0.001	1.22	1.15	1.29	2.52	0.113	0.91	0.81	1.02	-0.14	-10.08	< 0.001
Body Mass Index	2.56	0.110	1.00	1.00	1.01	3.22	0.073	0.99	0.99	1.00	0.00	-0.28	0.780
Lifetime Diet attempts	3.94	0.047	1.01	1.00	1.03	14.12	< 0.001	0.95	0.93	0.98	-0.09	-6.53	< 0.001
Total Autonomous Motivation	85.31	< 0.001	1.04	1.03	1.04	3.29	0.070	0.99	0.97	1.00	-0.04	-2.85	0.004
External motivation	1.44	0.231	1.01	0.99	1.03	2.11	0.146	1.02	0.99	1.05	0.00	0.27	0.791
Introjected motivation	28.41	< 0.001	1.04	1.02	1.05	0.19	0.667	0.99	0.97	1.02	-0.05	-2.97	< 0.001
Amotivation	282.55	< 0.001	0.84	0.82	0.85	3.60	0.058	0.96	0.92	1.00	-0.01	-0.39	0.699
Intrinsic aspiration only	72.45	< 0.001	1.26	1.20	1.33	6.81	0.009	1.15	1.03	1.27	0.00	0.15	0.885
Connection Hobbies	18.91	< 0.001	1.13	1.07	1.20	2.45	0.118	1.09	0.98	1.22	-0.01	-0.67	0.502
Sedentary Hobbies	5.40	0.020	0.95	0.90	0.99	1.37	0.242	0.95	0.87	1.04	-0.01	-0.72	0.475
Active Hobbies	2.85	0.091	1.04	0.99	1.09	4.81	0.028	1.10	1.01	1.20	0.00	-0.01	0.991
Arty Hobbies	18.75	< 0.001	1.12	1.06	1.18	4.36	0.037	1.11	1.01	1.22	0.00	-0.05	0.958
Food Hobbies	49.92	< 0.001	1.17	1.12	1.23	2.03	0.155	1.06	0.98	1.16	0.01	0.61	0.541
Mental Hobbies	0.12	0.731	1.01	0.96	1.06	28.29	< 0.001	1.32	1.19	1.46	0.03	1.75	0.081
Reading Hobbies	31.88	< 0.001	1.13	1.08	1.18	16.01	< 0.001	1.18	1.09	1.28	0.00	0.10	0.923
Travel Hobbies	23.35	< 0.001	1.12	1.07	1.17	6.03	0.014	1.12	1.02	1.22	0.01	0.84	0.404
SEIFA index of disadvantage	-	-	-	-	-	1.11	0.293	1.01	0.99	1.02	0.00	-0.05	0.958

Missing data for SEFIA cases removed listwise across variables

Supplementary analyses were used to compare motivation levels with weight loss between those who lost less than 5% (*n* = 2242), those who lost 5% but less than 10% (*n* = 2305) and those who lost 10% or more (*n* = 592) over the 12 weeks (Table 5). The observed differences represented a fraction of a standard deviation.

**Table 5:** Autonomous motivation and introjected motivation values between different weight loss groups based on total percentage lost over 12 weeks (*n* = 5139). Superscripts denote significant differences (*p*<.01) based on post-hoc Bonferroni comparisons

	0-4.9% loss *N* = 2242		5-9.9% loss *N* = 2305		10+% loss *N* = 592		Total *N* = 5176		ANOVA	
	M	SD	M	SD	M	SD	M	SD	F	*p*
Autonomous Motivation	15.42^a^	2.81	15.28^a, b^	2.74	15.03^b^	2.80	15.31	2.78	4.70	0.01
Introjected motivation	4.09^a^	1.60	3.86^b^	1.61	3.75^b^	1.60	3.94	1.60	16.84	< 0.001

## Discussion

Autonomous motivation is widely considered as an important construct for behaviour change, particularly around longer term, goal-orientated pursuits. The Diet Mindset Quiz was designed to measure motivation, life aspirations and intrinsic interests (hobbies), which were then examined across three stages of weight management – initiation which was defined as signing up to a program, early behavioural commitment which was defined as completing the initial 12 weeks of the program, and early success which was considered as percentage weight loss. In this large, ecologically valid sample of mostly female Australians living with overweight or obesity, the models explained minimal variance for each stage of weight management considered (1–5%). Although the overall predictive value was low, autonomous and introjected motivation prior to starting a program had a very small positive effect on the probability of signing up to the weight loss program, and a small negative effect on weight loss outcomes after 3 months for those who stayed on the program. Having broader intrinsic life aspirations increased the probability of signing up and completing the weight loss program. The pursuit of mentally orientated hobbies was among the strongest motivational predictors of being classified as a stayer although, again overall the predictors accounted for less than 2% of the total variance.

Despite the proposed theoretical importance of autonomous motivation for weight loss and health behaviour change, the current analysis did not find a strong or consistent association between autonomous motivation and weight loss success. Previous analyses reporting significant observations have generally been based on curated stages of observed motivation. For example, Webber et al. [15] observed motivation change over a 16-week web-based weight loss study but used motivation levels at week 4 to predict weight loss at week 16 in a small female sample. This explained 6% of the variance for weight loss without controlling for other variables. In contrast, the total model for percentage weight lost in our analysis predicted 5% of the total variance, which was primarily driven by gender and age effects. A separate weight loss study reported that 6-month motivation, and not baseline motivation, was moderately associated (beta 0.20) with weight loss [23]. These investigators applied a highly controlled in-person study and explored change in AM between time points before selecting a motivational variable for inclusion in their predictive model. These studies focus on early behavioural success, and it would be reasonable to predict that the association between early motivation would be strongest at the initiation stage. We failed to observe this. With so much variation in the way that motivation is combined and timing of measurements, it is unclear if the minimal effect sizes in our data are a result of measurement, timing or valid very small effects. Based on previous weight loss programs, it is also unclear whether the absence of formal motivational support in the TWD could explain absence of some of the lack of predictive value in later stages. Both of the previous studies mentioned above provided motivational support through personal education, regular appointments [23] and motivational interviewing [15]. Given the strong theoretical basis of SDT, studies with more regular assessments of motivation are needed in samples outside of clinical trials.

The better utility of the broader life aspirations and hobbies over motivational measures specific to behaviours may be also explained by the temporal sensitivity of capturing behaviour-specific motivation versus the relative stability of broader motivational contexts. Together, the findings and inconsistency in motivation throughout an intervention highlight the need for multiple assessments of motivational facets. The act of initiating a program itself alters motivational state - signing up builds momentum and shifts motivation prospectively - meaning that baseline assessment may capture motivation before any program-induced change has occurred. We did not quantify the time between the quiz and signing up, so this could also account for the reduced predictive potency.

Previous studies have reported that those with higher AM are more likely to achieve clinically meaningful weight loss of 5% of their initial body weight [15]. Our study found the opposite – those in the highest categories of weight loss had lower AM and introjected motivation. The effect sizes were small, represented a small increase in probability (3%) and a fraction of a standard deviation of actual difference in motivational scores across weight loss groups; therefore these offer limited practical relevance for those promoting health behaviour change. The size and change in direction of the effects of motivation observed here suggest AM needs clearer measurement or evaluation and further work is needed to establish the association between early AM and behavioural initiation. Approach motivation reflects the expectation of reward from pursuing a goal but does not necessarily indicate readiness to act on it. Those who were motivated but not yet ready may have engaged with the program differently, contributing to the apparently paradoxical finding [24].

More recent theories about SDT emphasise the difference between goal pursuit versus growth [25], suggesting that self-regulatory factors may be more important as people progress. Cybernetic motivation emphasises self-regulation through conscious goal setting, monitoring, reviewing and resetting; processes associated with program engagement behaviours (such as logging food intake and tracking weight). In contrast, organismic motivation reflects autonomous, growth-oriented goal pursuit [25]. What motivates a person to initiate a new goal may therefore be different from what sustains their behaviour once engaged [25, 26]. It is plausible that in the context of a structured 12-week program, cybernetic self-regulatory behaviours are more proximal determinants of weight loss than pre-program autonomous orientation, and that these may be partially independent of AM level at baseline. Consistent with this, program engagement is among the strongest known predictors of weight loss success in structured programs [20] and personal dispositions that are more goal-orientated have been associated with simple health behaviour change [27].

Despite modest magnitude, the current results suggest that understanding a person’s wider aspirational context could be relevant for increasing the probability of both starting and sustaining engagement with a weight loss program. The predictive value of intrinsic aspirations and hobby engagement may also reflect something broader than either construct captures alone. Using a measure similar to the aspirational goals outcome used in the current study, Vallerand et al. [28] reported global orientations that were more autonomously characterized positively predicted passion and engagement in hobbies. Therefore, there may be some association between the two. Minimal attention has been given to passion directly in the context of weight management, yet passion has also been noted to influence the way that people pursue and prioritise goals [29]. Further exploring broader intrinsic motivators and aspirations, and possibly passion, in terms of weight management initiate could provide interesting future direction.

### Strengths and limitations

A strength of the current analysis was the ability to explore initiation of behaviour change, which is rarely considered in previous studies. Capturing initiation is difficult but also critical to understand to support behaviour change [27]. A review of weight loss expectations concluded that evidence was too limited and discrepant [30]. A primary study exploring 47 women who signed up but never attended an initial treatment suggested a variety of outcomes such as dieting history and outcome evaluations could be used to profile women who were more ready for weight loss [31]. However, these data are based on the group that could be defined as starters in our analysis.

Our study was strong in terms of its ecological validity and sample size. We were able to consider a variety of variables in addition to motivational ones. However, participant characteristics were also biased due to reliance on self-selection - attracting a higher proportion of female participants typical for most weight management programs. The sizeable minority of males were included in analyses and gender only had predictive value for weight loss. The bias towards recruiting older women also likely represents greater interest in weight management. Overall, the sample had motivational profiles that were skewed toward favourable attributes, higher AM, engagement with hobbies and high intrinsic life aspirations. This also suggests possible bias introduced from self-selection, although there are no normative motivational profiles for comparison, and other studies including highly motivated samples have reported modest but stronger effects perhaps due to more homogeneously motivated samples.

There has been discussion about the utility of the measure of motivation used in this study and whether it should be a single or double construct [32]. We chose to factor analyse the tool to create a measure that fit our data which also aligned with general theory about AM but did not consider CM specifically. With a lack of consistency in the literature in the assessment of motivation, it becomes difficult to interpret results across studies. Our Quiz also assessed hobbies which have not previously been considered. Development of this measure was piloted but coding allowed for only presence of a hobby rather than the amount of engagement in specific hobbies which may have improved predictive value.

## Conclusions

Motivational theories are evolving to include broader constructs [24] and better real-world application [33]. Our study suggests that wider aspirations could be considered as potential contributors to initiation and staying on a weight loss program in future studies, but generally measurement and timing of assessing motivation requires a skilful brush. Overall, this research suggests that motivational variables could contribute to progress through a weight loss program, but that the effects are small and lack practical implication. Taken in the context of other findings on autonomous motivation, it may be less important to assess underlying motivations for a behaviour than to support individuals’ motivation as they seek to change their behaviour [11, 34].

## Data Availability

The datasets used and/or analysed during the current study are available from the corresponding author on reasonable request.

## Abbreviations

AM: Autonomous motivation
BMI: Body Mass Index
CM: Controlled motivation
CSIRO: Commonwealth Scientific and Industrial Research Organisation
SDT: Self-determination Theory
SEIFA: Socio-economic Indexes for Areas
TWD: Total Wellbeing Diet
